# Therapeutic Community Gardening as a Green Social Prescription for Mental Ill-Health: Impact, Barriers, and Facilitators from the Perspective of Multiple Stakeholders

**DOI:** 10.3390/ijerph192013612

**Published:** 2022-10-20

**Authors:** Carly J. Wood, Marie Polley, Jo L. Barton, Claire L. Wicks

**Affiliations:** 1 School of Sport, Rehabilitation and Exercise Science, University of Essex, Colchester CO4 3SQ, UK; 2 Marie Polley Consultancy Ltd., Hitchin SG4 0AP, UK; 3 School of Health and Social Care, University of Essex, Colchester CO4 3SQ, UK

**Keywords:** nature, mental ill-health, social prescribing, community, health, wellbeing

## Abstract

The UK government has invested £5.77 million in green social prescribing to prevent and tackle mental ill-health. Therapeutic community gardening, one type of green social prescription, provides a range of health outcomes. However, for increased accessibility, a greater understanding of how it impacts mental health and the facilitators and barriers to referral, uptake, and attendance by individuals with mental health problems is required. We conducted and thematically analysed interviews with thirteen stakeholders including social prescribing link workers and garden staff; and focus groups with twenty garden members. The mechanisms by which therapeutic community gardening were suggested to impact mental health were by engaging members with nature and the outdoors, providing hope for the future and facilitating social support and relationships. Factors facilitating referral, uptake, and attendance included a holistic and person-centred approach, which is flexible around health needs. Barriers included awareness of the full offering of therapeutic community gardens and accessibility, in terms of physical location and waiting lists. Given that nature-based interventions have the potential to protect and enhance population health and offer cost savings through reduced reliance on other health services; overcoming these barriers is key to ensuring that therapeutic community gardening is more widely available as an additional mental health treatment.

## 1. Introduction

In the UK it is estimated that one in four individuals experience at least one diagnosable mental health problem in any year [1]; whilst one in six experience a common mental illness such as depression at any time [2]. These rates are likely to be exacerbated by the coronavirus pandemic which has widened health inequalities, increased social isolation, loneliness, and mental ill-health [3,4,5,6]. Common treatment approaches for mental ill-health include medication such as anti-depressants, and psychological therapies such as Cognitive Behavioural Therapy (CBT) [7]. In 2017–2018, 7.3 million people, equivalent to 17% of the adult population, were prescribed anti-depressants; with 0.93 million being prescribed antidepressants continuously between April 2015 and March 2018 [8]. Given that anti-depressants are associated with withdrawal symptoms such as insomnia and suicidal ideation, their use should be carefully considered [8]. Furthermore, a meta-analysis by Fournier et al. [9] revealed that anti-depressants only provide significant positive effects compared to a placebo for severe depression. In relation to CBT, Johnsen and Friborg [10] found that its efficacy as a treatment has diminished in recent clinical trials, although this could be due to improper administration. Psychological therapies also have long waiting lists and are only available for limited time periods, typically between 5–20 sessions [11], impacting their ability to treat patients in times of need.

Social prescribing offers a means by which third sector organisations can provide non-medical sources of support to address mental, psychosocial, and socioeconomic needs [12]. Patients are referred to sources of support within their local communities, including activities such as walking, gardening and arts groups. Referrals are typically made by a social prescribing link worker who acts as the ‘link’ between the referring professional, which could include GPs and social workers, and community group; and discusses the patients’ needs with them before referring them to a suitable activity [12,13]. The National Health Service (NHS) has committed to diversifying the range of social prescriptions to ensure accessibility across the UK; aiming for over 900,000 people to be referred to schemes by 2023/24 [14].

Green social prescribing is one type of social prescription which aims to improve health and well-being through exposure to, and multisensory interaction with natural environments [15]. In July 2020 the UK government announced a £4 million cross-government investment aimed at preventing and tackling mental ill-health through green social prescribing; with additional investment released to take the total to £5.77 million [16]. Green social prescriptions consist of three main health-enhancing elements: (i) direct interaction with natural environments; (ii) social interaction and (iii) meaningful activity [15].

Therapeutic community gardening is a type of green social prescription which uses garden space and gardening activities to help people improve their mental health, build social skills, and develop confidence; with qualified therapist input or mental health support [17,18,19,20]. Individuals can be referred by a referring professional, or via self-referral; with professional input distinguishing therapeutic community gardening from non-therapeutic gardening activities such as community allotments. In addition to the three-core green social prescribing elements, therapeutic community gardening also promotes participation in moderate-intensity physical activity [21,22] and healthy eating [20]. Recent reviews and meta-analyses on the health benefits of therapeutic community gardening have demonstrated a wide range of health outcomes including reductions in depression, anxiety, and loneliness; and improvements in quality of life, life satisfaction, and community belonging [22,23,24]. However, these findings were not specific to individuals with defined health needs, nor were they focused on the use of therapeutic community gardening as a social prescription [23,24]. For therapeutic community gardening and green social prescribing to be upscaled and more widely available, a greater understanding of the facilitators and barriers to prescription and participation by individuals with mental ill-health is required, from multiple perspectives. Whilst studies in this area are limited, one recent qualitative study identified that effective referral pathways and clear marketing of green provisions are essential to the success of green social prescribing; and that there is a need for improved accessibility and inclusivity of services [25]. However, this study did not include the perspectives of service users.

The aims of this study were therefore to: (i) understand how therapeutic community gardening impacts the mental health of attendees and (ii) identify the barriers and facilitators to referral and uptake of community-based therapeutic gardening projects from the perspectives of multiple stakeholders.

## 2. Materials and Methods

### 2.1. Green Activity Provider: Trust Links

Trust Links is an independent mental health charity based in South Essex, UK which offers a range of services and support to members of the community experiencing mental ill-health, including ‘Growing Together’ (GT) which compromises four therapeutic gardening sites. GT is offered to adults (aged 18 years+) who have been referred to the gardens to support their mental ill-health, as well as their socialisation and onward progression. GT beneficiaries are referred to as ‘members’, creating an inclusive environment in which all attendees are equally valued. GT members experience a range of needs at varying levels of severity and include individuals with anxiety, depression, schizophrenia, bipolar disorder, personality disorder, neurodiversity, and mild learning disabilities. Referrals to GT are from a wide range of sources including GPs, community psychiatric nurses, social prescribing link workers, mental health teams, social workers, job centres, voluntary organisations and self-referral. Members attending take part in various gardening activities including sowing seeds, potting, and general garden maintenance. When on site, members are supported by horticultural project workers who lead gardening activities, support staff who support the mental health of members; and volunteers, who are members of the public recruited to support members, engage with the project, and maintain the gardens.

### 2.2. Recruitment

A range of stakeholders were invited to take part in semi-structured interviews via email or verbal invitation. Stakeholders were purposively selected based on their job role to ensure representation from all key personnel involved from referral through to session delivery and mental health support. Contacted stakeholders included primary care mental health staff, social prescribing link workers, and Trust Links volunteers and employees. Primary care mental health staff are individuals who provide specialist mental health support through organisations such as GP surgeries; whilst social prescribing link workers are individuals who receive patient referrals from primary care settings and discuss patients’ needs with them before referring them to suitable services within the community. Invited Trust Links staff members fulfilled a range of roles including coordinating garden activities, supporting member mental health, and running other Trust Links wellbeing services. Volunteers were individuals who were recruited to support members and garden activities. Trust Links provided the research team with contact details for stakeholders who might be interested in participating in an interview. Stakeholders were contacted by a member of the research team and provided with study information via a participant information sheet.

GT members were purposively selected to take part in focus groups based on which garden site they attended, their mental health diagnosis and length of attendance at GT to ensure representation across the garden sites. Invited members had also indicated their willingness to take part during consent procedures undertaken as part of additional research. GT members were invited to take part by GT staff. This approach was taken for three reasons; (i) coronavirus restrictions prevented research staff from regularly attending the gardens, (ii) staff were aware of members’ mental health status, duration of attendance and the garden/s attended, and (iii) as members were familiar with staff, it was hoped they would feel more comfortable declining the invitation if they did not wish to take part. Staff were provided with training on the aims of the research and recruitment procedures, including the importance of gaining consent.

All stakeholders provided informed consent prior to participation in the study. As stakeholders and members provided consent prior to the day of the interviews and focus groups, and in the case of members via GT staff, the research assistant confirmed consent immediately prior to the interview and focus groups. Ethical approval was granted by the School of Sport, Rehabilitation and Exercise Sciences Ethics Sub-committee at the University of Essex. The research assistant conducting the interviews and supporting the focus groups was trained in qualitative data collection techniques and analysis.

### 2.3. Interviews

Thirteen semi-structured interviews were conducted via Zoom (n = 10) or telephone (n = 3) between May-September 2021. Zoom interviews were recorded using Zoom software, whilst telephone interviews were recorded via Dictaphone. Interviews were conducted from a private space at the participants and researchers’ place of work or in their homes, lasting between 20 min and 1-h 23 min. All participants were asked about barriers and facilitators to referral and uptake of the service from the perspective of their role. Trust Links staff were asked additional questions about the impact on members’ health.

### 2.4. Focus Groups

One focus group was held outside at each of the GT sites in September 2021 and recorded using two Dictaphones. One focus group involved four members, two involved five members and one involved six members. The focus groups lasted between 1-h and 1-h and 31 min and were led by two peer researchers with lived experience of attending GT to support their mental health. It was felt that the inclusion of peer researchers would help members to feel more comfortable during the focus group process. The peer researchers received training on facilitating focus groups from a research assistant who provided support during the sessions. Members were asked questions about their experience of attending GT and the impact on their mental health.

### 2.5. Data Management and Data Security

Audio recordings were downloaded and saved on the University’s secure server in a password-protected folder only accessible to the research team. Interview and focus group recordings were transcribed verbatim and anonymised by a transcription company. All raw data was deleted in accordance with ethical requirements.

### 2.6. Analysis

Data were managed and coded using NVivo software version 12 (QSR International Pty Ltd., Doncaster, Australia, 2018). Data from interviews and focus groups were combined as questions in both the interviews and focus groups addressed the same topics. Transcripts were coded thematically [26]. Initially, two interview transcripts were coded independently by two authors and following discussion, a coding framework was developed and used to code the remaining transcripts. The coding framework was revised as coding continued and further codes emerged from the data. Themes emerged as coding progressed and were actively produced through discussions between the research team and the exploration of the data. Throughout the analysis, the researchers were aware that their different backgrounds were likely to influence the way they read and coded the data, especially as one author had extensive experience in social prescribing and another in the health benefits of nature. A-priori themes were used as a means of declaring each author’s potential bias to the data analysis as well as drawing on previous knowledge of barriers and enablers in this area.

As data analysis progressed and themes were actively developed, we discussed our own assumptions of the codes and themes against our own backgrounds, with each author being able to question their own and each other’s assumptions. The researcher with the least experience in social prescribing and nature-based research carried out the primary analysis to also ensure that there was the least bias in the coding of the data. In the final stage of the analysis, three overarching themes were identified which reflected the aims of the research. A combined deductive and inductive approach was therefore used throughout the analysis.

## 3. Results

### 3.1. Participants

All stakeholders (100%) approached to take part in an interview consented to participate. Participants included Trust Links staff members (n = 5), garden volunteers (n = 5), social prescribing link workers (n = 2), and a primary care mental health team member (n = 1). Seven of the participants were female (53.8%), including both social prescribing link workers, the primary care mental health team member, two Trust Links volunteers and two employees. The remaining three Trust Links volunteers and the remaining three staff members were male.

Twenty garden members agreed to take part in focus groups, six of whom were female (30%). GT members had a range of mental health diagnoses, with their duration of attendance ranging from 4 months to 8 years.

Three overarching themes reflecting the aims of the research were identified, including; ‘Impact on mental health’ which describes potential mechanisms through which engaging with GT can enhance mental health, ‘Facilitators to referral, uptake and engagement’ which describes factors that facilitate engagement with the service from the perspective of multiple stakeholders, and ‘Barriers to referral, uptake and engagement’ which describes factors that may prevent engagement with the service from the perspective of multiple stakeholders.

### 3.2. Impact on Mental Health

Three sub-themes emerged from the impact on mental health overarching theme: nature and the outdoors, hope for the future and social support and relationships.

#### 3.2.1. Nature and the Outdoors

Members described how undertaking gardening tasks and nurturing plants offers a distraction from their mental illness, and that being in the gardens is an opportunity to let off steam. Members described interacting and connecting with nature through visual, audio, olfactory, and haptic means. Connecting with nature was described as enjoyable and gave members’ a sense of pride and purpose, for example being involved in the process from sowing to harvest and observing wildlife benefitting from their work.

*“After we [have] done it, we sit here and where we feed all the birds they come and they like to peck at all the ground and eat all the feed, and it’s nice to see the wildlife enjoying what all the members and volunteers have done”.*

(Member)

Members also spoke of moments where noticing nature directly affected their mental health by helping them to put life into perspective. When feeling down, something as small as looking at a flower could help reframe members thinking and help them to realise that things might not be as bad as they thought. A social prescribing link worker described noticing small aspects of nature as a form of mindfulness which they felt was beneficial to mental health. A staff member spoke of how the diversity of the spaces allows members to find solace in the gardens in various ways, whether the distraction offered through tasks, or migrating from the main group to find a quiet area for reflection.

*“…I think the garden is quite important for that, for how they [members] deal with their problems, so I think that’s positive. Because they’re very different as well in terms of how the space is used either to work in or just simply sit down, in the herb garden”.*

(Staff member)

#### 3.2.2. Hope for the Future

Staff described how members are often lonely and isolated or lack confidence and self-esteem when they join. They shared how members may not have felt able to make choices about various aspects of their lives and lived in ‘survival’ mode where they were unable to thrive. One staff member described how members could be a blank canvas when they arrived at GT.

*“Well just that you know sometimes members come along and they’re like a blank canvas and we’re there to show them good things that they can put onto that blank canvas”.*

(Staff member)

Staff described how the various activities available helped members to build confidence, and social and practical skills, giving them a sense of worth, purpose and belonging. A member of staff reflected that increased happiness and confidence allowed members to see more opportunities in life, take risks, learn new things, and push themselves. Both members and staff acknowledged that attending GT may not lead to full recovery, but that the support helps them to understand more about their mental health, and develop self-management strategies and resilience, enabling them to move forward.

*“… to give them the tools to be able to succeed and what Trust Links does is it allows small steps and they represent the building blocks for their foundation. So the more little steps the deeper their foundation and it keeps them upright in the future but they might have relapses but they might not be bad relapses that they had in the past”.*

(Staff member)

Staff described members’ achievements beyond GT which included finding employment or volunteering opportunities, developing personal relationships, and returning to education. A member of staff shared that these achievements might be ‘down the line’ for members, but members are actively working towards these goals. Members described personal ambitions for the future, such as returning to employment, undertaking qualifications, or having their own allotment.

*“And I’d like to try to work up to a job one day maybe, get back into the job again like I used to. I used to be a Team Leader in my last job, and when that went after 20 years it got stressful”.*

(Member)

#### 3.2.3. Social Support and Relationships

Members and staff spoke about how members’ shared experience of mental ill-health helps to create a sense of belonging and a positive non-judgemental space for recovery. Members were able to share their skills, experience, and knowledge with each other and with staff. Members valued the social connections made, which offered friendship and social support.

*“We get more [support] from each other than we, this place you get more from each other than you do from them [staff]. I’m not saying they don’t do a good job, but we get more from each other”.*

(Member)

In some cases, members’ existing social connections were causes of, or associated with their mental ill-health and attending GT set them on the ‘straight and narrow.’ Staff shared how some friendships moved beyond GT. A member of staff spoke about how members felt able to be their ‘authentic’ selves at the gardens and that this facilitated relationships.

*“Being yourself, you can actually, you’re not frightened to be yourself, you’re not going to be stigmatised by being yourself. And actually, if you learn that you like, start liking yourself then you might have a chance of actually facing other people. But it’s actually getting to learn to like yourself”.*

(Member)

Staff and members spoke of the positive relationship between GT and the local community, with residents supporting the gardens through donations, shop purchases and attending events. Staff believed these activities helped members connect with the community and gave members a sense of pride. Members developing social capital was also evident through their accounts of taking part in community volunteering, fundraising events, and helping neighbours.

*“Being valued, isn’t it? And feeling you have a part in the community. And with our project if you’re members of the public, you can come and walk around the garden and, you know, that kind of gives you a lot of—they [members] feel proud of the garden, yes. We’re not just doing it for ourselves, you’re doing it for everybody…”*

(Staff member)

Social prescribing link workers and GT staff believed that the non-traditional approach of therapeutic community gardening helps to engage harder to reach populations who may not engage with traditional therapy, such as young people and men. They described men ‘opening up’ when working shoulder to shoulder in the gardens and not being expected to ‘spill their guts’, but instead having a casual chat. Younger people were also thought to be more likely to engage with the informal setting and outdoor activities.

### 3.3. Facilitators to Referral, Uptake and Engagement

Two sub-themes emerged from the facilitators to referral, uptake and engagement overarching theme: holistic approach and flexibility and inclusivity.

#### 3.3.1. Holistic Approach

Social prescribing link workers spoke of the multiple health-enhancing elements of GT as factors that encouraged referral. The opportunities to make social connections and feel a sense of community belonging were two key factors that supported referral, along with opportunities for physical activity, being in nature and exposure to fresh air and vitamin D.

*“…the social aspect of it, a lot of the social aspect is a lot of my patients are quite socially isolated or feeling very low because they’re very isolated by their diagnosis. So, you know, knowing that they can connect with other people in that way and have peer support is really, really helpful”.*

(Link worker)

GT takes a holistic approach to supporting members with various aspects of their lives. Staff and members described opportunities for developing life skills, training opportunities and support in areas such as employment, funding and benefits, physical health, and relationship issues. Staff also shared how GT helps members access other healthcare services, which they felt in the long-term reduces the reliance on other services. Two members disclosed how they had been able to come off medication since attending.

*“… indirectly the NHS should be paying this place to actually keep it going because it is saving them so much money. Because all of us would be probably knocking on the Doctor’s door, “Give us pills, give us pills, give us pills”.*

(Member)

#### 3.3.2. Flexibility and Inclusivity

Staff and members described how the service is person-centred and offers flexibility including choice around which activities members engage with and no fixed duration of attendance.

*“There’s always a role that someone can play. No matter how complex you are. No matter who you are. Any details about you. You know, we have a space here for everyone, and that’s through how much use we have. Yes. I would say that is through how many activities we have and how abundant they are”.*

(Staff member)

Staff also spoke of some members choosing to take on responsibility for specific roles, such as mowing lawns or managing the shops. Some members described how their engagement would vary depending on how they felt on a given day and that they felt welcomed regardless of their preferences or ability.

*“I think I feel with the disability that I could come and I could just sit and drink coffee with the people that don’t do the gardening or you know, that option is available. You don’t feel stigmatised for doing that, nobody minds if that’s what you want to do”.*

(Member)

Staff described how GT has a flat hierarchical structure. Staff do not wear uniforms or name badges creating a sense of equality.

*“…from our structural—there’s no us and them. There’s no staff and members. You know, we don’t wear- there’s a reason we don’t wear uniform. We don’t wear lanyards. We eat lunch together. We have our coffees together”.*

(Staff member)

Staff spoke of some rules and processes that are in place to facilitate inclusivity and help support members’ mental health including bad language being prohibited, progress reviews and checking in with non-attending members.

### 3.4. Barriers to Referral, Uptake and Engagement

Two sub-themes emerged from the barriers to referral, uptake and engagement overarching theme: awareness and accessibility.

#### 3.4.1. Awareness

Many stakeholders commented on the need to raise awareness of GT. Social prescribing link workers wanted to know more about the full range of activities and support available to help them to ‘sell’ and match individuals to the service. This was felt to be particularly important for individuals who do not have a pre-existing interest in gardening, perceive gardening as hard work, have uncertainty about how they might fit in, do not recognise how gardening can benefit their mental health or have previous negative social experiences.

*“…perhaps to give us more of an overview about some of the projects and how we can make them sound more appealing to some of the patients because I think sometimes it’s all in the wording isn’t it and I try and talk to people about things in a way that I hope will make it sound quite an attractive proposition. But I think if we don’t know the exact ins and outs of it [the service] then it’s harder to, harder to sell really”.*

(Link worker)

Similarly, staff and garden volunteers felt that raising awareness of what GT offers with referring professionals and the wider community was essential to supporting as many people as possible. One volunteer emphasised the importance of raising awareness of self-referral, while another volunteer felt that for individuals being referred the lack of awareness or ‘unknown’ aspect of joining the gardens was a difficult barrier to overcome. The volunteer shared that once members were through the gates, the person-centred approach was vital to ensuring members felt comfortable and allowed them to settle in at their own pace.

#### 3.4.2. Accessibility

The limited number of places at GT was a barrier to referral, especially when link workers knew individuals would be placed on a waiting list. Another barrier was the physical location of the gardens, particularly if individuals do not drive or have mobility issues. Volunteers and staff spoke about members relying on family members to provide transport and occasions when members had shared negative experiences of using public transport. Members shared details of long or difficult journeys sometimes involving multiple buses or trains to reach the gardens. Staff and volunteers also described how symptoms of mental ill health made it more difficult for members to use public transport. Social prescribing link workers said this could result in referral to services which were geographically closer.

*“I think as soon as you get somebody with anxiety or depression the thought of getting there is a problem, the thought of coming out the house is a problem, the thought of getting on a bus or a train or anything is a problem and then meeting people they don’t know is a problem. So it’s, while the services out there are great it’s getting people to go to them that’s the thing, the difficulty”.*

(Link worker)

Staff spoke about individuals being at different stages of recovery and that it had to be the right time to access GT, which in some cases might not be until an individual reached a particularly low point. Once engaged with the service, it was still difficult for some members to attend regularly or take part in all the activities. Staff and members described how declines in mental health, changes to medication or failure to take medication could cause members to disengage. When members disengage, members are not removed from the service. Instead, staff contact members and support them to return to the gardens when they are ready to reengage. One member spoke of concerns around upsetting other members as a barrier to regularly attending but was aware that their mental health status negatively affected their perception of social encounters.

*“… I sometimes think I’ve done wrong and it stops me coming again because I think to myself, I shouldn’t have done that, shouldn’t have done that”.*

(Member)

Whilst GT strives to be an inclusive environment, members with physical health conditions described how tasks could exacerbate issues. For one member, their inability to engage fully in gardening tasks and the recognition that their condition was degenerative gave them the ‘hump’.

*“Sometimes it’s worth the risk of having a bad back for two weeks, just to get out of the house and do something I enjoy doing. Sometimes it’s difficult when you want to do something and your physical problems mean you are going to suffer afterwards for, but sometimes you do take that risk”.*

(Member)

Staff and members shared that additional barriers included members’ having other commitments, such as moving into employment or family commitments and the cold weather. One member said that they did not enjoy being outside in the winter, while another shared that they would rather ‘wrap up’ than be stuck indoors.

## 4. Discussion

This study sought to (i) understand how therapeutic community gardening impacts the mental health of attendees and (ii) identify the barriers and facilitators to referral and uptake of community-based therapeutic community gardening projects from the perspectives of multiple stakeholders. The three main mechanisms through which GT appears to benefit members’ well-being are the opportunities to engage with the natural environment, offering hope for the future, and the development of social relationships and support. Furthermore, the holistic approach, flexibility and inclusivity of GT facilitate referral, uptake and continued engagement by members. Members receive support for all aspects of their health and well-being and have the freedom to use the service in a personal way. Two key barriers to referral, uptake and engagement were a lack of knowledge of the complete offering of the service and the accessibility of the gardens including the physical location and the number of places available.

### 4.1. Mechanisms of Wellbeing Benefits

The benefits of exposure to the natural environment for health and well-being are widely recognised as evidenced by the investment into green social prescribing by the UK Government [16]. The premise that the natural environment enhances psychological health through stress-reducing or psychologically restorative spaces has been proposed by well-established theories of nature and health [27,28]. In keeping with these theories, we found that spending time in the gardens and engaging in gardening offered distraction and respite from stressors and enhanced positive affective states. These findings are supported by previous studies investigating the benefits of community and allotment gardening [21,29,30,31]. Some members shared that they had been able to stop taking medication, while others stated that they preferred to manage their mental health through spending time at the gardens, rather than taking medication. This demonstrates the effectiveness of therapeutic community gardening as an additional treatment option for mental ill-health and helps to combat the issue of overprescribing highlighted by Taylor et al. [8], which has positive cost and carbon benefits [32].

A reciprocal relationship between members and nature was evident, whereby members nurtured plants and wildlife and were nurtured in return, experiencing enjoyment, sense of purpose and improved well-being. This reciprocal relationship may be explained through increased nature connection [33], which is associated with increased well-being and pro-environmental behaviour [34]. Further, pro-environmental behaviour is associated with increased life satisfaction [35,36]. Therapeutic community gardens are therefore beneficial to both human health and the natural environment; contributing to Governmental policies concerned with improving mental well-being [37] and environmental protection [38].

Hope is associated with improved outcomes of CBT [39], increased quality of life [40] and reported as an outcome of gardening for both healthy and clinical populations [23]. Members expressed hope for the future through personal ambitions, including employment and education. Related outcomes through which gardening may help foster a sense of hope include increased self-esteem, confidence, and positive thinking [22] reduced stigma and negative stereotypes [41] and vocational achievements [42]. Staff also described how developing a sense of hope might protect against relapse which is consistent with hope conferring protective benefits against the development of mental health disorders [43,44].

Many individuals with mental ill-health have unmet social needs leading to loneliness [45], with loneliness exacerbating conditions [46]. GT offers opportunities to build social relationships and experience peer support, in a non-judgemental space where members feel able to be their authentic selves. Community gardens have been found to encourage openness [29], reduce stigma [47], and help to develop positive social identities [41], with improved social networks and reduced isolation improving mental health outcomes [22]. Furthermore, therapeutic community gardens have also been found to improve community well-being through increased social interaction, quality of life, community growth and social capital [22,48]. Thus, increased provision of therapeutic community gardening is likely to benefit the communities in which they are integrated. In line with the NHS’ Personalised Care Model [49], this demonstrates the potential of therapeutic community gardens to benefit the well-being of the wider population, in addition to an estimated 5% with complex needs, and 30% with long-term health conditions. York and Wiseman [50] state that community garden members can become ‘social agents of change’ through their involvement in growing and selling fresh produce to the wider community and sharing the gardens through community events, emphasising community development as a determinant of health.

### 4.2. Facilitators of Prescription and Attendance at Growing Together

The holistic approach of GT emerged as a key facilitator for uptake and engagement and is in keeping with the principles of social prescribing [16]. Link workers described the opportunities for physical activity as important when making referral decisions. Individuals with mental ill-health are at increased risk of developing co-morbid physical health conditions [51,52,53], which physical activity can prevent or manage. Furthermore, GT supports multiple health outcomes by engaging participants in meaningful physical activity that involves direct interaction with nature [15]. Whilst gardening is the primary function of GT, the gardens provide opportunities for healthy eating, skill development, and social connections [20,21,22,31]. Green social prescriptions such as therapeutic community gardening offer non-medical sources of support to address mental, psychosocial, and socioeconomic needs [12]. With around 20% of GP appointments primarily due to social rather than medical needs [54], there is an increased need for holistic services that can address a range of health determinants.

GTs person-centred approach offers flexibility in how members access and use the service and inclusivity. Members can find space in the gardens for solace or choose to engage in gardening or group activities. Therapeutic community gardens offer freedom and flexibility for individuals to gain what is needed from attending on any given day while feeling part of something bigger [29]. Members are welcome to attend for as long as they need the service; with numerous members staying on to volunteer. The flexibility and continuity enable members to use the gardens with their changing needs and align with the NHS’ personalised care model, which aims to support the health of the entire population as they move through various levels of need [49]. In contrast, traditional treatments are offered for limited durations [11], and do not support the wider population’s well-being. Whilst the unlimited attendance at GT may require continued funding, nature-based interventions have been reported to offer per-person cost savings through avoided costs on public health and other services, such as GPs, A&E, and police services, in the region of £830 after 1 year and £6450 after 10 years, demonstrating their cost-effectiveness [55]. However, these figures are estimated based on one programme and do not incorporate the cost savings due to reduced loneliness and increased life satisfaction. Thus, the actual cost savings may be greater.

### 4.3. Barriers of Prescription and Attendance at Growing Together

Staff and volunteers spoke of the importance of raising awareness of GT to ensure continuing success and growth. Efforts are made by the organisation to engage with external services and the community, however, a lack of knowledge about the service by some link workers impacted referral decisions and made it difficult to give potential members enough information about what attendance at GT would involve. This finding is in line with the work of McHale et al. [25]; who identified a need for established referral pathways and clear marketing of green social prescriptions. Social prescribing link workers are a critical link between referring professionals and social prescribing activities in the community [7]. Training for social prescribing link workers, including qualifications [56] and time to build relationships with local organisations is essential for the success of social prescribing and supporting the person-centred approach of social prescribing. Organisations could be encouraged to provide information about their services in accessible formats (e.g., Infographics) to aid busy link workers. For many individuals with mental ill-health the barriers described in this study intersect, leading to insurmountable barriers without additional support. For example, living with the symptoms of mental ill-health and reliance on public transport can make it impossible for some to make it through the gates [7,25]. The location of the gardens is also a key barrier to initial engagement with GT. Solutions were posed including offering a bus service and a support or buddy system for new members. Developing relationships with individuals being referred prior to first attendance may also help alleviate anxieties.

While GT strives to be an inclusive environment, there are opportunities to develop inclusivity through co-produced and outreach activities. This would benefit individuals with limited mobility or physical health conditions who cannot participate fully. Ensuring minority community groups are targeted through outreach work and that diverse images are used in marketing campaigns may help reduce health inequalities. Additional barriers included members having other commitments and lack of funding. Offering extended opening hours at weekends may help those who need the service around other commitments. For organisations with limited resources, weekends could be offered as a ‘lighter touch’ service for individuals experiencing milder symptoms or in recovery, but who may still benefit from therapeutic community gardening. It is hoped the growing evidence base around the effectiveness of therapeutic community gardening and nature-based interventions more widely, together with demonstrable cost-effectiveness may support increased funding opportunities and a shift to nature-based interventions becoming as commonly prescribed as other medicalised options.

### 4.4. Strengths and Limitations of the Research

The findings of this study represent the perspectives of multiple stakeholders involved with the GT at different stages and in different contexts. Peer researchers were employed to lead the focus groups to facilitate engagement and open conversation. Thus, the study has ensured that the views of individuals involved with the gardens in various ways are represented. However, the study has numerous limitations. While members were informed that their participation would not affect their use of the service, some members declined to take part in the focus groups and may not have wanted to discuss their experience due to concerns over service removal. Some members may also have declined or not been asked to take part due to their mental illness or a relapse in their recovery. Thus, the views of garden members included in this study may not represent the views of all members. The authors also acknowledge that an opportunity for participants to provide feedback on the findings would have ensured that the views of individuals who did take part were adequately captured. Furthermore, only two social prescribing link workers participated in the research and as such the full range of barriers or facilitators of link workers who worked with different systems and processes may not have been captured. Whilst this research is based on the GT therapeutic community gardens, it is acknowledged that other therapeutic gardening projects may have different or additional barriers. Future research should therefore incorporate the perspectives of individuals referred to a range of green social prescriptions and therapeutic community gardens in order to explore the impact, barriers, and facilitators to these activities more widely and inform strategies to upscale provision and referral.

## 5. Conclusions

This research explored the experiences of a range of stakeholders involved in the GT therapeutic community gardening projects to support upscaling and more widespread availability of therapeutic community gardening activities via social prescriptions. The opportunities for social interaction and peer support, engagement with the natural environment and wider community, as well as hope for the future are suggested as pathways through which attendance enhances mental health. The results highlight the importance of taking a holistic approach enabling members to tailor the use of the service to their individual needs, facilitating continued engagement and supporting the health of the whole person rather than just their diagnosis. Barriers that social prescribing link workers, individuals or organisations need to overcome relate to knowledge of the range of services offered by therapeutic community gardens, factors relating to the mental health of an individual and accessibility of gardens. The experience of members and volunteers, and the positive view of the benefits of therapeutic community gardening demonstrate the acceptability of therapeutic community gardening as a social prescription. Therapeutic community gardening and nature-based interventions more widely have the potential to protect and enhance population health, build healthy and connected communities, and offer considerable cost savings through reduced reliance on other health and social care services.

## Data Availability

The data presented in this study are available via the UK Data Service Repository on request from the corresponding author.

## References

[1] Mental Health Taskforce (2016). The five year forward view for mental health. Mental Health Taskforce.

[2] McManus S.B.P., Jenkins R., Brugha T. (2016). Mental Health and Wellbeing in England: Adult Psychiatric Morbidity Survey 2014.

[3] Marmot M., Allen J., Goldblatt P., Herd E., Morrison J. (2020). Build Back Fairer: The COVID-19 Marmot Review. The Pandemic, Socioeconomic and Health Inequalities in England.

[4] Heron P.N., Spanakis P., Crosland S., Johnston G., Newbronner E., Wadman R., Walker L., Gilbody S., Peckham E. (2021). Loneliness among people with severe mental ill health during the COVID-19 pandemic: Results from a linked UK population cohort study. medRxiv.

[5] Jaspal R., Breakwell G.M. (2020). Socio-economic inequalities in social network, loneliness, and mental health during the COVID-19 pandemic. Int. J. Soc. Psychiatry.

[6] Kwong A.S.F., Pearson R.M., Adams M.J., Northstone K., Tilling K., Smith D., Fawns-Ritchie C., Bould H., Warne N., Zammit S. (2021). Mental health before and during the COVID-19 pandemic in two longitudinal UK population cohorts. Br. J. Psychiatry.

[7] Aughterson H., Baxter L., Fancourt D. (2020). Social prescribing for individuals with mental health problems: A qualitative study of barriers and enablers experienced by general practitioners. BMC Fam. Pract.

[8] Taylor S., Annand F., Burkinshaw P., Greaves F., Kelleher M., Knight J. (2019). Dependence and Withdrawal Associated with Some Prescribed Medicines: An Evidence Review.

[9] Fournier J.C., DeRubeis R.J., Hollon S.D., Dimidjian S., Amsterdam J.D., Shelton R.C., Fawcett J. (2010). Antidepressant drug effects and depression severity: A patient-level meta-analysis. JAMA.

[10] Johnsen T.J., Friborg O. (2015). The effects of cognitive behavioral therapy as an anti-depressive treatment is falling: A meta-analysis. Psychological Bulletin.

[11] NHS England Overview–Cognitive Behavioural Therapy (CBT) 2018. https://www.nhs.uk/mental-health/talking-therapies-medicine-treatments/talking-therapies-and-counselling/cognitive-behavioural-therapy-cbt/overview/.

[12] Chatterjee H.J., Camic P.M., Lockyer B., Thomson L.J.M. (2018). Non-clinical community interventions: A systematised review of social prescribing schemes. Arts Health.

[13] Kimberlee R. (2015). What is social prescribing?. Adv. Soc. Sci. Res. J..

[14] NHS England (2019). Universal Personalised Care: Implementing the Comprehensive Model.

[15] Robinson J.M., Breed M.F. (2019). Green Prescriptions and Their Co-Benefits: Integrative Strategies for Public and Environmental Health. Challenges.

[16] NHS England Green Social Prescribing n.d. https://www.england.nhs.uk/personalisedcare/social-prescribing/green-social-prescribing/.

[17] Crouch D. (1992). British allotments: Landscapes of ordinary people. Landscape.

[18] Gross H., Lane N. (2007). Landscapes of the lifespan: Exploring accounts of own gardens and gardening. J. Environ. Psychol..

[19] Veen E.J., Bock B.B., Van den Berg W., Visser A.J., Wiskerke J.S.C. (2016). Community gardening and social cohesion: Different designs, different motivations. Local Environ..

[20] Howarth M., Brettle A., Hardman M., Maden M. (2020). What is the evidence for the impact of gardens and gardening on health and well-being: A scoping review and evidence-based logic model to guide healthcare strategy decision making on the use of gardening approaches as a social prescription?. BMJ Open.

[21] Park S.-A., Shoemaker C., Haub M. (2008). Can Older Gardeners Meet the Physical Activity Recommendation through Gardening?. HortTechnology.

[22] Wakefield S., Yeudall F., Taron C., Reynolds J., Skinner A. (2007). Growing urban health: Community gardening in South-East Toronto. Health Promot. Int..

[23] Soga M., Gaston K.J., Yamaura Y. (2017). Gardening is beneficial for health: A meta-analysis. Prev. Med. Rep..

[24] Spano G., D’Este M., Giannico V., Carrus G., Elia M., Lafortezza R., Panno A., Sanesi G. (2020). Are Community Gardening and Horticultural Interventions Beneficial for Psychosocial Well-Being? A Meta-Analysis. Int. J. Environ. Res. Public Health.

[25] McHale S., Pearsons A., Neubeck L., Hanson C.L. (2020). Green Health Partnerships in Scotland; Pathways for Social Prescribing and Physical Activity Referral. Int. J. Environ. Res. Public Health.

[26] Braun V., Clarke V. (2006). Using thematic analysis in psychology. Qual. Res. Psychol..

[27] Ulrich R.S., Simons R.F., Losito B.D., Fiorito E., Miles M.A., Zelson M. (1991). Stress recovery during exposure to natural and urban environments. J. Environ. Psychol..

[28] Kaplan R., Kaplan S. (1989). The Experience of Nature: A Psychological Perspective.

[29] Bailey A., Kingsley J. (2020). Connections in the garden: Opportunities for wellbeing. Local Environ..

[30] Genter C., Roberts A., Richardson J., Sheaff M. (2015). The contribution of allotment gardening to health and wellbeing: A systematic review of the literature. Br. J. Occup. Ther..

[31] Wood C.J., Pretty J., Griffin M. (2016). A case-control study of the health and well-being benefits of allotment gardening. J. Public Health.

[32] Department of Health and Social Care (2021). Good for you, good for us, good for everybody. A Plan to Reduce Overprescribing to Make Patient Care Better and Safer, Support the NHS and Reduce Carbon Emissions.

[33] Zylstra M.J., Knight A.T., Esler K.J., Le Grange L.L.L. (2014). Connectedness as a Core Conservation Concern: An Interdisciplinary Review of Theory and a Call for Practice. Springer Sci. Rev..

[34] Maund P.R., Irvine K.N., Reeves J., Strong E., Cromie R., Dallimer M., Davies Z.G. (2019). Wetlands for Wellbeing: Piloting a Nature-Based Health Intervention for the Management of Anxiety and Depression. Int. J. Environ. Res. Public Health.

[35] Laffan K. (2020). Handbook on Wellbeing, Happiness, and the Environment. Green with Satisfaction: The Relationship between Pro-environmental Behaviours and Subjective Wellbeing.

[36] Netuveli G., Watts P. (2020). Pro-environmental behaviours and attitudes are associated with health, wellbeing, and life satisfaction in multiple occupancy households in the UK Household Longitudinal Study. Popul. Environ..

[37] HM Government (2018). A Green Future: Our 25 Year Plan to Improve the Environment.

[38] H.M. Government (2021). Net Zero Strategy: Build Back Greener.

[39] Gallagher M.W., Long L.J., Richardson A., D’Souza J., Boswell J.F., Farchione T.J., Barlow D.H. (2020). Examining Hope as a Transdiagnostic Mechanism of Change Across Anxiety Disorders and CBT Treatment Protocols. Behav. Ther..

[40] Wu H.C. (2011). The protective effects of resilience and hope on quality of life of the families coping with the criminal traumatisation of one of its members. J. Clin. Nurs..

[41] Mason J., Conneeley L. (2012). The Meaning of Participation in an Allotment Project for Fathers of Preschool Children. Br. J. Occup. Ther..

[42] Clatworthy J., Hinds J., Camic P. (2013). Gardening as a mental health intervention: A review. Ment. Health Rev. J..

[43] Rydén A., Karlsson J., Sullivan M., Torgerson J.S., Taft C. (2003). Coping and distress: What happens after intervention? A 2-year follow-up from the Swedish Obese Subjects (SOS) study. Psychosom. Med..

[44] Long L.J., Gallagher M.W., Gallagher M.W., Lopez S.J. (2018). Hope and Posttraumatic Stress Disorder.

[45] Meltzer H., Bebbington P., Dennis M.S., Jenkins R., McManus S., Brugha T.S. (2013). Feelings of loneliness among adults with mental disorder. Soc. Psychiatry Psychiatr. Epidemiol..

[46] Mushtaq R., Shoib S., Shah T., Mushtaq S. (2014). Relationship between loneliness, psychiatric disorders, and physical health? A review on the psychological aspects of loneliness. J. Clin. Diagn. Res..

[47] Fieldhouse J. (2003). The Impact of an Allotment Group on Mental Health Clients’ Health, Wellbeing and Social Networking. British Journal of Occupational Therapy.

[48] Firth C., Maye D., Pearson D. (2011). Developing “community” in community gardens. Local Environ..

[49] NHS England Comprehensive Model of Personalised Care 2018. https://www.england.nhs.uk/publication/comprehensive-model-of-personalised-care/.

[50] York M., Wiseman T. (2012). Gardening as an Occupation: A Critical Review. Br. J. Occup. Ther..

[51] Yu M., Zhang X., Lu F., Fang L. (2015). Depression and Risk for Diabetes: A Meta-Analysis. Can. J. Diabetes.

[52] Sher Y., Lolak S., Maldonado J.R. (2010). The impact of depression in heart disease. Curr. Psychiatry Rep..

[53] Pinquart M., Duberstein P.R. (2010). Depression, and cancer mortality: A meta-analysis. Psychol. Med..

[54] The Low Comission (2015). The Role of Advice Services in Health Outcomes: Evidence Review and Mapping Study.

[55] Pretty J., Barton J. (2020). Nature-Based Interventions and Mind-Body Interventions: Saving Public Health Costs Whilst Increasing Life Satisfaction and Happiness. Int. J. Environ. Res. Public Health.

[56] Groves K. Education standards, competency framework, accreditation, and qualifications. Proceedings of the 4th Annual International Social Prescribing Conference.

